# Association between changes in pain or function scores and changes in scapular rotations in patients with subacromial shoulder pain: a prospective cohort study

**DOI:** 10.1186/s40945-022-00143-4

**Published:** 2022-08-15

**Authors:** Zohreh Jafarian Tangrood, Gisela Sole, Daniel Cury Ribeiro

**Affiliations:** Centre for Health, Activity, and Rehabilitation Research (CHARR) – School of Physiotherapy, University of Otago, Dunedin, New Zealand

**Keywords:** Subacromial shoulder pain, Scapular rotations, Scapular locator, Association, NPRS, PSFS

## Abstract

**Background:**

Scapular dyskinesis is reported as one of the potential factors contributing to the presentation of pain in subacromial shoulder pain. In clinical practice, the evaluation and control of scapular dyskinesis is considered important for managing the subacromial shoulder pain. The aim is to determine the association between changes in pain or function and changes in scapular rotations in participants with subacromial shoulder pain.

**Method:**

Pain, function and scapular rotations were measured in 25 participants with subacromial shoulder pain at baseline and after 8 weeks. Pain was measured with Numeric Pain Rating Scale (NPRS) and function was measured with Patient Specific Functional Scale (PSFS). Scapular rotations were measured with a scapular locator at 60°, 90° and 120° of scapular arm elevation. Spearman rank correlations (r_s_) were used to assess the association between variables.

**Findings:**

No association was observed between changes in pain or function scores with changes in scapular upward/downward rotations (r_s_ = 0.03 to 0.27 for pain and − 0.13 to 0.23 for function) and scapular anterior/posterior tilt (r_s_ = − 0.01 to 0.23 for pain and − 0.13 to 0.08 for function) of arm at 60°, 90° and 120° elevation. Data associated with scapular internal/external rotation was not reported due to low reliability.

**Conclusion:**

These findings reject associations between changes in pain or function scores and scapular rotations. Future observational study is warranted using a multifactorial approach to understand potential factors that contribute to the presentation of subacromial shoulder pain.

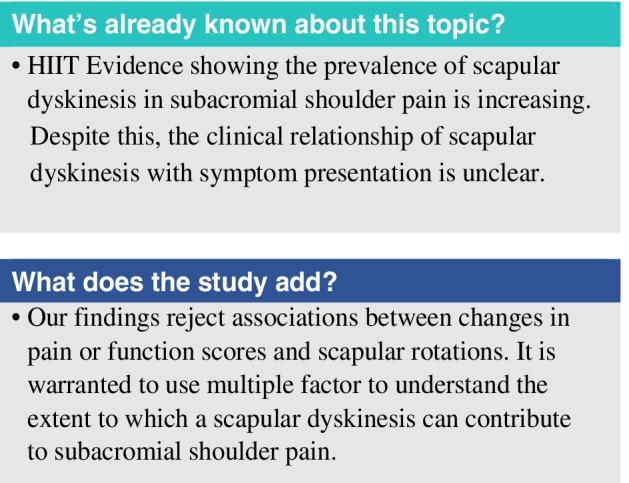



## Introduction

Exploring normal scapular rotations is essential for understanding normal movements of the shoulder joint, as well as for understanding whether abnormal scapular movement causes or is affected by shoulder pain [1]. At rest, the scapula presents with 4° to 5° of upward rotation, 35° to 41° of internal rotation and 10° to 13° of anterior tilt relative to the thorax [1–4]. During arm elevation, the scapula progressively rotates upward, and tilts posteriorly [5–7]. Scapular internal-external rotation during arm elevation is a bit more complex [5, 7, 8]. The scapula rotates internally with the arm elevation up to 120°. Once arm elevation moves further than 120° the scapula rotates externally [5, 8]. Different ranges were reported for scapular rotations during arm elevation. Overall, at 120° the arm elevation, studies reported that the scapula rotates a range from 20° to 48° upwardly, from 3° to 18° posteriorly, and from 1° to 12° internally [9–13].

Scapular dyskinesis has been defined as increased or decreased scapular upward rotation, increased scapular internal rotation, and decreased posterior tilt [4, 8, 14–16]. The number of studies indicating scapular dyskinesis in patients with shoulder pathologies is increasing, however, it is still unclear if scapular dyskinesis is caused by other factors contributing to shoulder pain or is a trigger for shoulder degenerative disorders [14, 17]. A review of observational studies on the prevalence of shoulder pain injuries in athletics with scapular dyskinesis showed a trend towards shoulder injury despite not having a significant statistical association [18]. These findings indicate that scapular dyskinesis could not be a sole risk factor for shoulder injuries. Also, there is insufficient evidence to support the view that the scapula adopts a specific pattern of movement in patients with subacromial shoulder pain [19, 20]. The findings of one observational study only showed that athletes who developed shoulder pain in 2 years demonstrated a significant lower scapular upward rotation at lower degrees of arm elevation [21]. Therefore, more investigations are needed to seek the clinical relevance of scapular dyskinesis with subacromial shoulder pain.

Subacromial shoulder pain is described as pain at the lateral shoulder joint spreading between the neck and elbow that worsens during arm movements, especially during arm elevation [22, 23]. Inconsistent results were reported by cross-sectional studies that assessed pain or function scores between patients with subacromial shoulder pain with scapular dyskinesis and patients without scapular dyskinesis [16, 24–26]. One such study reported that patients with scapular dyskinesis had higher functional ability [24], which is counter-intuitive for clinical practice. In contrast, other studies reported higher functional impairments [25] or similar pain or functional scores [16, 26, 27] for patients with scapular dyskinesis compared to those with normal movement patterns. Findings of some laboratory studies indicated that changes in pain influenced scapular rotations [28, 29]. For example, experimentally induced subacromial pain caused increased scapular upward rotation in asymptomatic participants [28] or using lidocaine injection led to increased scapular internal rotation in patients with subacromial shoulder pain [29]. A limitation of laboratory-based studies is that changes in pain (i.e., induced increase or decrease pain) happens without changing in other accompanying factors. For example, reduced pain is not because of improving in rotator cuff muscle strength (that may affect scapular orientation). Therefore, these studies did not include possible effects of structural changes or injury (such as rotator cuff tears) on pain, nor consider psychosocial factors that may contribute towards patients’ pain experiences [28, 29].

Findings of our previous study showed an association between improvement in function and decrease in scapular dyskinesis over 8 weeks follow-up [30]. However, that study used a clinical test to assess scapular movement pattern and that method is limited when the observer is meant to distinguish subtle scapular dyskinesis from normal scapular rotation [16, 25]. One way of avoiding limitations from that previous study is to measure scapular movement patterns using quantitative methods.

The scapular locator is considered as an acceptable, reliable and valid instrument for measuring scapular movement [31–36]. This tool requires palpation of the root of the spine, the acromion angle of scapula and the inferior angle [32]. Palpating bony landmarks of scapula during arm elevation in cadavers has been shown to have the accuracy of 0.67 cm for palpating the root of spine, 0.98 cm for palpating the acromion angle, and 0.46 cm for palpating the inferior angle of scapula [37]. Using a scapular locator as a palpation-based method for locating scapular landmarks has been reported to be a fast, easy and well standardized method of measuring actual scapular rotations [37, 38].

The primary aim of this study was to assess the association between changes in pain or function with changes in scapular rotations in patients with subacromial shoulder pain over an 8-week period. The secondary aim was to determine the changes in pain, and function in the cohort of patients with subacromial shoulder pain over the period.

## Methods

### Design

This was an observational, prospective, cohort study following participants with subacromial shoulder pain for 8 weeks. The study followed the Strengthening and Reporting of Observational Study (STROBE) guidelines [39]. Ethics approval was obtained from the University Ethics Committee [Reference H17/080].

### Setting

Participants with subacromial shoulder pain were recruited from 1st September 2017 until the 30th February 2018 from the local and University community via emails and notice board flyers. They were assessed at the Biomechanics Laboratory, at the Centre for Health, Activity and Rehabilitation Research (CHARR), School of Physiotherapy, University of Otago, Dunedin, New Zealand. Eligible participants were provided with an informed written consent form prior to taking part in the study.

### Participants

#### Inclusion and exclusion criteria

Participants were included if they were 18 years old or more with shoulder pain. They were assessed based on the British Elbow and Shoulder Society (BESS) guidelines [40] and were included if they had one positive finding of the following tests [41]: (1) painful arc movement during shoulder flexion or abduction, or (2) pain with the Jobe’s test [40], or (3) pain on resisted lateral rotation or abduction [42].

Participants with a history of shoulder dislocation, or subluxation, shoulder surgery or cervical surgery within the last 6 months were excluded. Furthermore, participants with symptoms of inflammation or systemic diseases, signs of paresthesia in the upper extremities, hemiplegic shoulder pain, suspected frozen shoulder, or positive clinical signs of full thickness rotator cuff tear and signs of pain in acromioclavicular joint were excluded. Clinical signs of full thickness rotator cuff tears were a positive external rotation and internal rotation lag tests [43, 44]. Clinical sign of acromioclavicular joint pain was pain on palpation and at the end range of arm elevation [23]. Participants with bilateral shoulder pain were assessed on the dominant side [9].

### Variables

#### Demographics

Participants’ demographic characteristics were collected at baseline. These included age, sex, weight, height, self-reported hand dominance, shoulder pain side, the shoulder pain duration, previous shoulder pain injuries, and treatment for their shoulder pain.

#### Pain

Positional shoulder pain was measured when participants held their arm at 60°, 90° and 120° scapular arm elevation using the numeric pain rating scale (NPRS) [45]. This scale has 11 score ratings from 0 to 10, with 0 indicating no pain and 10 being the most severe pain. In this scale, a change of 2 points represents the minimal clinically important difference (MCID) [46, 47]; a change of 2 to 3 points was considered to be as ‘medium’ or ‘meaningful change; and a change ≥ 3.5 to 4 points as ‘significant’ change [48, 49].

#### Function

The Patient Specific Functional Scale (PSFS) was used for scoring individual-specific functional limitations [50]. This tool has high construct validity to differentiate between patients who present clinical improvements and those who do not [51], and high discriminant validity to identify low, medium and high functional disabilities [49]. The PSFS has moderate to high reliability for participants with stable symptoms over time [51] . Participants were asked to name three to five activities that they were unable to perform or had difficulty due to their shoulder pain. For each functional activity, they were asked to rate the difficulty from 0 to 10 where ‘0’ indicates inability for performing the activity, and ‘10’ indicates ability to perform the activity as the same as before shoulder pain/injury. The total score rates from 0 to 10 where a greater score indicates better function. A 1.3-point change represents a MCID; 2.3-point change represents a medium, and greater than 2.7-point change a large clinical change [49].

#### Scapular rotations

**Fig. 1:**
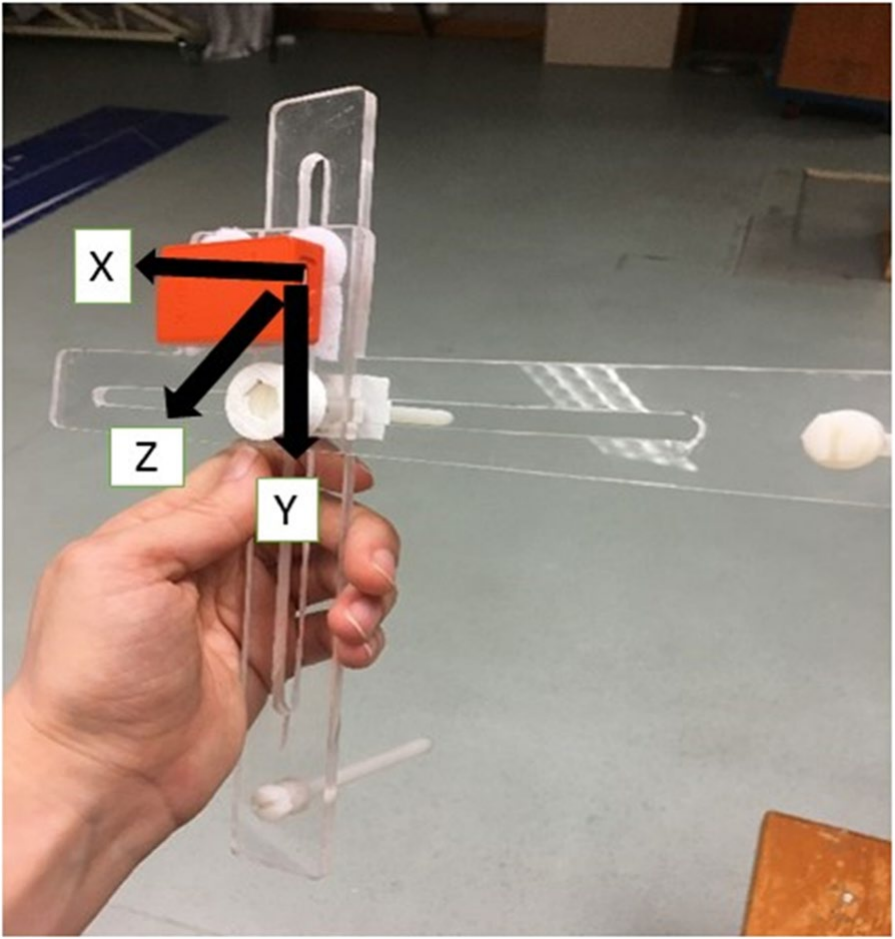
Scapular locator with an inertial sensor (orange) attached to the vertical arm

Scapular locator was used to measure scapular upward/downward rotations at coronal plane, and scapular anterior/posterior tilt at sagittal plane. The scapular locator was a custom-built tool comprising two crossing transparent plastic arms with three pins. The plastic arms are joined by a central bolt allowing for adjustment of the three pins over the individual’s bony anatomical landmarks [52] (Fig. 1). Our previous study indicated that scapular locator measurements had good to excellent reliability for scapular upward/downward rotations and scapular anterior/posterior tilt (ICC ranging from 0.73 to 0.93 of arm at 60°, 90° and 120° arm elevation) [36]. The standard error of measurement (SEM) and the smallest detectable differences (SDD) with 95%CI for scapular upward/downward rotation and scapular anterior/posterior rotation are presented in Table 1. We found poor reliability for measuring scapular internal/external rotations (ICC ranging 0.37 to 0.62). Given the low reliability reported for scapular internal/external rotation, we did not report the findings in that plane in this study [36] .

**Table 1:** Between-day reliability, standard error of measurement (SEM), and smallest detectable difference (SDD) when measuring scapular rotations during arm elevation with inertial sensor

Scapular kinematic	Scapular plane Arm position	ICC (3,1)	95% CI	SEM [degree]	SDD [degree]
Upward/downward rotation	60°	0.73	0.37 to 0.89	4.8	13.5
	90°	0.89	0.71 to 0.95	5.1	14.2
	120°	0.93	0.84 to 0.97	6.6	18.5
Anterior/posterior rotation	60°	0.80	0.51 to 0.91	3.9	10.9
	90°	0.86	0.67 to 0.94	4.3	12.0
	120°	0.87	0.71 to 0.95	5.6	16.5

Abbreviation: *ICC* intraclass correlation coefficient, *95% CI* 95% confidence interval for the ICCs

Three wireless inertial sensors (Xsens Technologies, NL) were fixed to the skin, using double-sided tape, on thorax, upper arm and on the scapular locator to measure scapular rotations. The thoracic sensor was placed on the center of the sternum with the Y axis pointing laterally, Z axis pointing anteriorly, and X axis pointing superiorly. The upper arm sensor (considering the right-hand side) was placed at middle third of arm, slightly posteriorly with the Y axis pointed medially, the Z axis pointed posteriorly and the X axis pointed superiorly. A Neoprene elastic cuff also was wrapped around the arm sensor to minimize soft tissue artifacts. The scapular locator sensor was positioned on the vertical bar of the scapular locator with the X axis pointing medially, the Y axis pointing inferiorly and the Z axis pointing posteriorly (Fig. 1). Data recorded from the inertial sensor was collected at a sampling frequency of 100 Hz in the Xsens MT manager (IMw_Pro_iseos, 2012, Xsens Technologies, NL).

### Measurement procedures

**Fig. 2:**
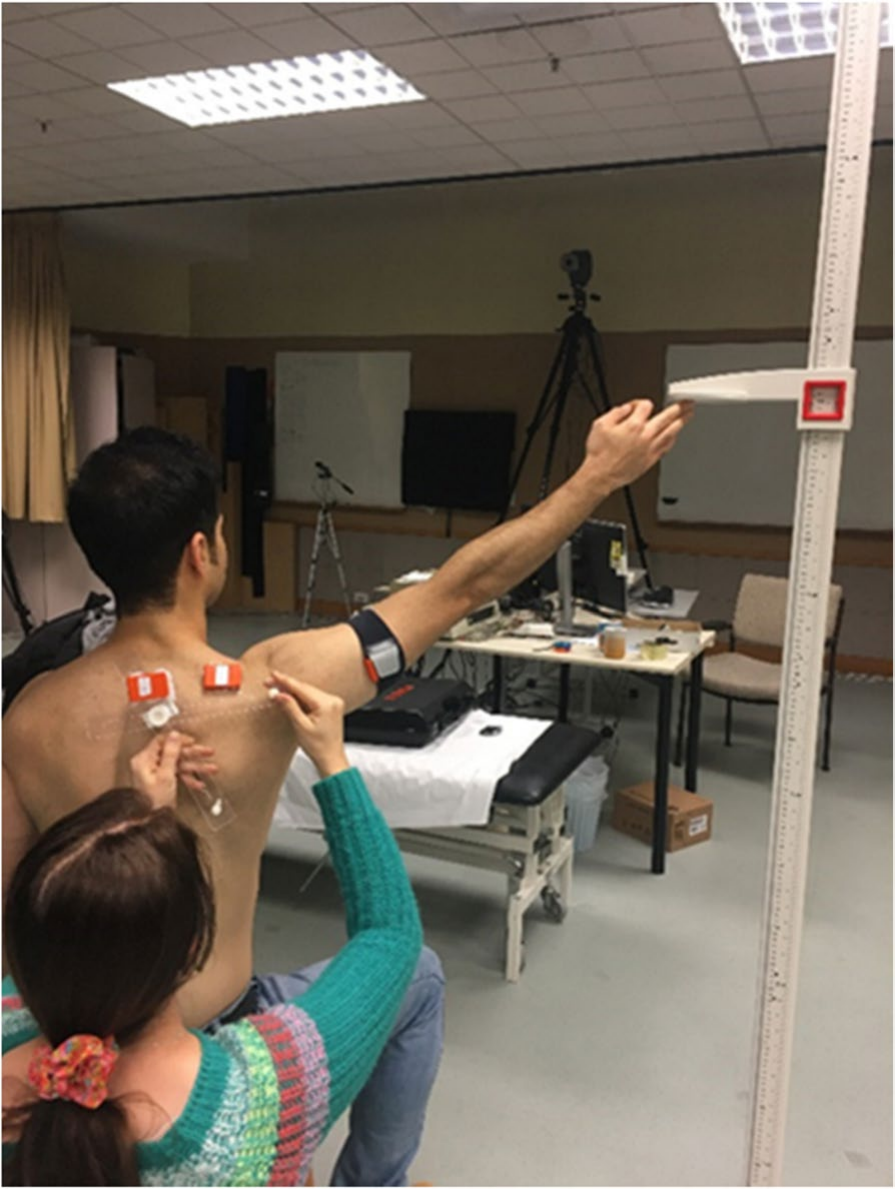
Scapular measurement on the painful side. The pole was used to guide the degree of arm elevation

For anatomical calibration of the sensors, recording was performed when participants sitting upright position, with the arm resting in anatomical neutral position and with the thumb pointing forward. An assessor, visually inspected such alignment. Scapular rotations were recorded when participants hold their painful arm at rest and 60°, 90°, and 120° in the scapular plane, with the elbow extended and thumb facing upward. To standardize arm positions, one upright pole with a movable target fixed on it, was placed at 30° in front of the coronal plane with respect to participant’s position (Fig. 2). A goniometer was used for setting up the upright pole in 30° anterior to the frontal plane passing through acromioclavicular joint. The assessor used a goniometer to adjust the arm in a respective angle and then asked participants to hold their arm with the target at the required position (i.e. 60°, 90°, and 120°). They were asked to keep head and trunk movements to a minimum measurement [12, 53]. In each angle, participants, first were asked to report their positional pain and then the assessor recorded the scapular positions by locating the scapular locator’s pins on the posterior acromial angle, the root of scapular spine, and the inferior scapular angle [32, 34]. Measurement was performed 3 times for each degree of arm elevation and was recorded for 5 seconds [32, 34]. Participants were able to rest between trials to avoid fatigue [53].

### Time points

Participants were assessed at two time points: at baseline and 8 weeks later. This period was selected as findings of a previous study recommended a minimum of 8 weeks is required to allow significant improvements in pain or function if patients with subacromial shoulder pain are treated with physiotherapy [54]. As it was an observational study, we did not offer any intervention to participants during the period of data collection. Participants could, if they wished to do so, seek care from healthcare professionals.

### Sample size estimation

Given no previous study assessed the correlation between pain or function scores and scapular rotations, when estimating the sample size required for this study, we had to make few assumptions. We considered relevant to have sufficient power to identify whether changes in pain or function scores could be explained by a change of, at least, 33% in scapular rotation. In other words, we considered relevant a coefficient of determination of 0.33, which converts to a correlation coefficient of 0.57. We set power at 0.8, considered a two-sided test with alpha set at 0.05, a correlation coefficient of 0.57 (or greater). Based on that, the smallest number required to detect a correlation of 0.57 (i.e., coefficient of determination of 0.33) was 22 participants. We did not make assumptions about the correlations between repeated measures over time. Hence, our sample size estimation is conservative. The sample size was estimated using the “ICC. Sample Size” package in R software [55].

### Data processing

Data from the inertial sensor located on the scapular locator were processed relative to the sensor on the sternum (thorax) for calculating scapular rotations. The orientation of the scapula was decomposed using the Euler sequence of Y-Z’-X” [56]. Rotation around the Y axis was referred to as “scapular internal/external rotation,” rotation around the Z’ axis as “scapular upward/downward rotation,” and rotation around the X” axis as “scapular anterior/posterior tilt.” The motion of the thorax was decomposed related to the global coordinate system. The orientation of the thorax was decomposed using the Euler sequence of ZXY axes: rotation around the Z-axis referred to lateral flexion, rotation around the X-axis referred to flexion-extension, and rotation around the Y-axis referred to axial rotation. Coding for extracting the raw data was written in MATLAB R2016b (The Mathworks Inc., Natick, MA). All data were entered in a Microsoft™ Excel 2013 spreadsheet. Scapular rotations around Y, Z’ and X” axes were calculated for each of the three repetitions and an overall mean angle was then calculated for each time point (i.e., baseline and follow-up). Positive direction in scapular rotations refers to scapular internal rotation, scapular upward rotation and scapular anterior tilt.

### Statistical analysis

Descriptive statistics were calculated using IBM SPSS statistics 25 [57] for continuous data including scapular rotations for each arm position (scapular upward/downward rotation and anterior/posterior tilt, pain (pain at different arm degrees) and PSFS at baseline and follow-up. In the case of missing data at follow-up, scores were entered based on multiple imputation [58]. For all inferential analyses, alpha was set at 0.05.

We used the change in scores (between baseline and follow-up) when assessing the correlation between pain or function and scapular rotations. Spearman rank correlation (r_s_) was used as it was expected a non-linear relationship between changes in pain or function and scapular rotations at 60°, 90° and 120° scapular arm elevation. At each degree of arm elevation, the relationships between changes in pain or function were explored with changes in scapular rotations. The strength of associations between variables was interpreted based on the following criteria: correlation coefficient of 0.25 or less was considered low; 0.26 to 0.50, considered as fair; 0.51 to 0.75, considered as good; and greater than 0.76, considered strong correlation [59].

We compared scores between baseline and follow-up for each outcome measure (i.e., pain, function, and scapular rotations). Paired t tests were used for assessing the difference between baseline and follow-up for all continuous data. When comparing changes at follow-up from baseline (X = follow-up –baseline) for pain or scapular rotations, a positive difference indicated increase in pain, increase in scapular upward rotation, and increase in scapular anterior tilt. When comparing changes for function scores at follow-up from baseline, a positive difference indicated improvement in function. Such information was used to determine whether changes in scores were greater than the minimum clinically important difference for each outcome measure.

## Results

### Demographic characteristics

Fifty-three participants showed interest in this study. Eighteen participants did not meet the inclusion criteria and 10 eligible participants withdrew from the study. Overall, 25 eligible participants (16 women, and 9 men ranging between 24 to 86 years) agreed to participate in the study. Demographic and clinical characteristics of recruited participants at baseline and the follow-up time point (8 weeks), and the difference in mean values between the two time points are presented in Tables 2 and 3. Two participants presented with bilateral shoulder pain. In those cases, we collected data for their dominant side. Shoulder pain duration ranged from 0.5 to 384 months with five participants reported acute shoulder pain (≤ 3 months) and 20 participants presented with chronic shoulder pain. Eight participants reported previous shoulder pain episodes, and 6 of them reported seeking treatment for their shoulder disorder. Out of 25 recruited participants at baseline, 9 participants were identified with obvious scapular dyskinesis, 13 with ‘subtle’ scapular dyskinesis, and three with ‘normal’ scapular rotation. At follow-up session, five participants (3 women and 2 men) dropped-out reporting reasons: being busy (*n* = 2), being out of town for unexpected reasons (*n* = 2) or being unwell at the time of follow-up (*n* = 1). Therefore, pain, function and scapular rotations were assessed in 20 participants at follow-up.

**Table 2:** Demographic and clinical characteristic of participants with shoulder pain at baseline (*N* = 25)

	Mean (SD)	Range
Age (years)	45.8 (13.8)	24 to 86
Weight (kg)	76.0 (15.2)	46.8 to 100
Height (cm)	169.2 (9.4)	153 to 195
BMI (kg/m^2^)	26.9 (4.8)	18.5 to 35.9
Female sex N (%)	16 (64%)	
Shoulder pain duration (months)(med)	12	0.5 to 384
Acute and subacute shoulder pain (< 3 months), N (%)	5 (20%)	
Chronic shoulder pain (> 3 months), N (%)	20 (80%)	
Hand dominance right side N (%)	Right side: 21 (84%)	
Affected side N (%)	Right side: 14 (56%) Left side: 11 (44%)	
Previous history of shoulder pain N (%)	8 (32%)	
Previous treatment of the shoulder	6 (24%)	

*Abbreviation*: *N* number of participants, *BMI* Body Mass Index

**Table 3:** Scapular rotations, pain and function at baseline and follow-up time points and the different between two time points (*N* = 25)

	Baseline mean and (SD)	Follow-up mean and (SD)	Mean Difference (95% CI)	***P***-value
**Scapular upward rotation**				
**60°**	8.4 (7.1)	6.6 (9.8)	- 1.8 (−6.2 to 2.5)	0.400
**90°**	18.0 (8.8)	17.2 (10.9)	- 0.8 (−5.7 to 4.1)	0.750
**120°**	27.3 (8.6)	23.3 (14.9)	−4.0 (−8.9 to 1.0)	0.112
**Scapular posterior tilt**				
**60°**	−0.4 (6.1)	−0.8 (6.1)	− 0.3 (−3.0 to 2.30)	0.790
**90°**	−2.4 (11.8)	0.7 (12.8)	3.1 (−1.6 to 7.9)	0.187
**120°**	−5.3 (18.1)	1.6 (19.6)	6.9 (0.2 to 13.7)	0.042*
**NPRS**				
**60°**	2.8 (2.2)	1.7 (1.5)	−1.1 (−2.0 to −0.2)	0.022*
**90°**	3.5 (1.9)	2.6 (1.8)	−0.9 (−1.5 to − 0.2)	0.013*
**120°**	3.9 (2.2)	2.8 (1.9)	−1.1 (− 1.9 to − 0.2)	0.018*
**PSFS**	45.7 (17.6)	54.8 (24.5)	9.2 (2.2 to 16.1)	0.012*

*NPRS* Numeric Pain Rating Scale, *PSFS* patient specific functional scale

^*^statistically significant difference (*P*-value ≤0.05). Mean difference was calculated as follow-up – baseline. Positive sign indicates increase in pain, scapular upward rotation, and scapular anterior tilt and increase in functional ability

### Association between changes in pain or function with scapular rotations

No association was observed between changes in pain or function scores and changes in scapular upward/downward rotations (r_s_ = 0.03 to 0.27 for pain, and r_s_ = − 0.13 to 0.23 for function) and scapular anterior/posterior tilt (r_s_ = − 0.01 to 0.23 for pain and r_s_ = − 0.13 to 0.08 for function) of arm at 60°, 90° and 120° elevations (Table 4).

**Table 4:** Correlation coefficients between changes in pain or function with scapular rotations

Arm degree	Upward/downward rotation (r_s)_	*P*-value	Anterior/posterior rotation (r_s)_	*P*-value
NPRS				
60°	0.03	0.888	- 0.01	0.968
90°	0.27	0.190	0.23	0.268
120°	0.15	0.472	0.09	0.666
PSFS				
60°	0.23	0.271	- 0.05	0.808
90°	- 0.13	0.548	0.08	0.718
120°	- 0.09	0.659	- 0.13	0.540

*Abbreviation*: *NPRS* Numeric Pain Rating Scale, *PSFS* patient specific functional scale, *p*-value ≤0.05

## Discussion

We assessed the association between changes in pain or function with changes in scapular rotations over 8-weeks follow-up in participants with acute and chronic subacromial shoulder pain. Our hypothesis was to observe an association between increased scapular upward rotation and improved pain or function scores. This study demonstrated no association between changes in pain or function scores and changes in scapular upward/downward rotation, and scapular anterior/posterior tilt.

### Association between changes in pain or function with scapular rotations

No significant associations were observed between changes in pain or function with scapular upward/downward rotation and anterior/posterior tilt measured with the scapular locator. This finding is consistent with the findings of a previous study assessing if change in pain could lead to change in scapular rotations in participants with full thickness rotator cuff tear [29]. That findings showed that an immediate reduction in pain (following lidocaine injection) resulted in no changes in scapular upward/downward rotation and anterior/posterior tilt [29]. Christiansen, Møller [26], also indicated no difference in functional scores over time between patients with subacromial shoulder pain group with and without scapular dyskinesis. The findings of this study, however, are inconsistent with two previous studies that indicated increased scapular dyskinesis, using the visual dyskinesis test, was associated with more functional disability [25, 30].

The inconsistency in findings from the literature and our study could be attributed to different methods used for assessing scapular dyskinesis. Unlike our study, previous studies used the scapular dyskinesis test and showed an association between scapular dyskinesis and functional deficit [25, 30]. The method used in previous studies had lower validity as they used scapular dyskinesis test that has limited reliability and validity compared with methods assessing 3-D measurement of scapular rotations [60–63]. The scarcity of studies measuring scapular kinematic using motion capture system [64], and the limitations of clinical tests for assessing scapular dyskinesis [14] have limited our understanding about the clinical role of scapular dyskinesis in patients with shoulder disorders.

In our study, most participants did not receive specific intervention, however, they reported different scores in pain and function. This indicates that participants pain and function may not be reflected their real changes. It is possible that psychological factors affect the presentation of pain and function scores. For example, patients with psychological distress report higher scores for pain [65], therefore future studies are recommended to consider a significant margin for changes in pain and function scores as one requirement of such assessments.

Inadequacy in identifying other contributing factors does not allow us to understand in which situation a scapular dyskinesis contributes to symptom presentation. For example, scapular dyskinesis is frequently reported in asymptomatic overhead athletes [66] or was highly associated with manual work tasks (e.g., amount of weight handled, time necessary to complete a given task, and the level of hand force exerted, etc.) in asymptomatic office workers [67]. In our study, we noticed scapular dyskinesis is obvious for participants with long duration of shoulder pain. Therefore, it is recommended future studies assess the association of these factors in presentation of scapular dyskinesis in patients with subacromial shoulder pain. Scapular dyskinesis should be seen in the global picture of patients’ profile to assess the efficacy of other contributing factors on scapular and symptom presentation.

### Clinical implications

The lack of a significant association between changes in pain with scapular rotation may be due to the relatively low levels of pain reported by the participants in this study at baseline (mean NRPS < 4/10) and small changes in pain (~ 1/10 across the 8 weeks). Low to moderate shoulder pain is common in patients with subacromial pain [68, 69]. Other studies exploring the influence of interventions (scapula-focused exercises or manual therapy) on scapular kinematics, pain and function in patients with subacromial pain syndrome, found that improvement for pain and function were not explained by changes in scapular kinematics [70–72]. Those findings, thus, support our study.

Altered scapular rotation may be one of multiple factors that participate in presentation of subacromial shoulder pain [73] or it may be presented as a result of an adaptive process. For example, scapular dyskinesis was reported higher in the dominant shoulder side than the non-dominant side, and in athletic tennis players than non-athletic players [21, 74]. Future studies should control for other contributing factors (e.g., shoulder pain duration, physical work demand, motor control impairment, comorbidity, previous neck pain) when assessing the association between scapular dyskinesis and pain or function in this population. Identifying these factors may clarify how much an altered scapular rotation could be considered a disorder in subacromial shoulder pain or a variation from normal scapular rotation [17].

### Limitations

Participants in our study did not present important clinical changes in pain or function scores from baseline to follow-up. It is possible that the duration of follow-up (i.e., 8 weeks) was not sufficient for changes in pain or function scores to occur. Those changes did not occur because most participants did not receive any form of treatment from healthcare professionals. Only 5 participants received treatments including medication, home-exercise, and soft tissue massage therapy.

Changes in scapular rotations from baseline to follow-up were insignificant and often located within SEM limits. Future studies are recommended to use reliable tools for measuring scapular rotations. Recent evidence indicated an excellent reliability using scapulometer when measuring scapular internal/external rotation and anterior/posterior tilt [75]. This could be a possible appropriate tool for future studies.

Five participants (20%) dropped-out and could have biased our estimates. However, we believe this limitation was minimized since we followed best practices and used multiple imputation to complete the dataset. We also acknowledge that other individual factors were not controlled for, and could be potentially confounding factors when analyzing the association between scapular rotations and pain or function. For example, symptom duration, age, sex, pain catastrophizing, and physical demands in daily life [76] may influence both scapular dyskinesis and pain or function. Therefore, any future cohort study should control potential confounders.

## Conclusion

Findings of this study demonstrated no association between changes in pain or function scores with changes in scapular upward/downward rotation and scapular anterior/posterior tilt. It is important to highlight that participants did not present minimal clinical changes in pain and function scores during the follow-up period. Additionally, it is warranted to use multiple factors to understand the extent to which a scapular dyskinesis can contribute to subacromial shoulder pain.

## Data Availability

The datasets used and/or analyzed during the current study are available from the corresponding author on reasonable request.
